# Vital Predictive and Prognostic Roles of Triglyceride‐Glucose Index in Women With Acute Myocardial Infarction: A Retrospective Cohort Study

**DOI:** 10.1002/hsr2.70157

**Published:** 2024-10-27

**Authors:** Xiao‐xia Qiu, Yong‐li Chen, Xin‐kang Wang, Re‐hua Wang

**Affiliations:** ^1^ Department of Cardiology Shengli Clinical Medical College of Fujian Medical University Fuzhou China; ^2^ South Branch of Cardiology Department Fujian Provincial Hospital Fuzhou China; ^3^ Department of Cardiology Shengli Clinical Medical College of Fujian Medical University Fuzhou Fujian China; ^4^ Fuzhou University Affiliated Provincial Hospital Fuzhou China; ^5^ Department of Electrocardiographic Diagnosis Fujian Provincial Hospital Fuzhou Fujian China; ^6^ Department of Cardiology Fujian Provincial Hospital Fuzhou China

**Keywords:** acute myocardial infarction (AMI), female, insulin resistance (IR), major adverse cardiac and cerebral events (MACCEs), triglyceride‐glucose index (TyG index)

## Abstract

**Background and Aims:**

As a biomarker of insulin resistance (IR) in patients with acute myocardial infarction (AMI), the triglyceride‐glucose index (TyG index) has received significant attention. However, most research on AMI has focused on male patients, as it is traditionally believed to primarily affect males. Therefore, this study was conducted on a female population with AMI to investigate the potential correlation between the TyG index and their outcomes.

**Methods:**

A total of 320 women who were admitted to Fujian Provincial Hospital for AMI between January 2017 and December 2019 were included in this study. The TyG index was calculated using the following formula: ln [fasting triglycerides (TG) (mg/dL) × fasting plasma glucose (FPG) (mg/dL)/2]. The primary endpoint of the study was the occurrence of major adverse cardiovascular and cerebrovascular events (MACCEs), which included all‐cause mortality, myocardial infarction, repeat revascularization, rehospitalization for heart failure and stroke. The association between the TyG index and unfavorable outcomes in female patients was investigated using the Cox proportional hazards regression model.

**Results:**

It was ultimately estimated that 111 patients developed MACCEs. Females with high TyG indices had a higher prevalence of diabetes, elevated heart rates, and hemoglobin A1c, as well as a higher likelihood of undergoing thrombus aspiration and stent placement. The TyG index was found to be positively correlated with the prevalence of hypertension, diabetes, low‐density lipoprotein cholesterol, hemoglobin A1c, and damaged vessels. However, this correlation was modest, yet statistically significant. Furthermore, after adjusting for conventional risk factors, the TyG index (HR: 4.292, 95% CI: 2.784–6.616, *p* < 0.001) was independently associated with MACCEs.

**Conclusion:**

As an independent risk predictor, the TyG index has the potential to enhance clinical outcomes for women with AMI.

## 1 Introduction

It is estimated that AMI accounts for a large portion of all deaths worldwide [1]. AMI have an increasing mortality rate in China [2]. Concomitantly, gender disparities in the mortality rate due to AMI are coming into focus. It was shown in an American epidemiological report that more women than men die from AMI within 1 or 5 years after their first episode [3]. There are several common clinical risk scoring systems for predicting the rate of postoperative mortality or postoperative adverse cardiovascular events in patients with AMI. The European System for Cardiac Risk Evaluation (EuroSCORE II) is mainly used to assess in‐hospital mortality. The SYNTAX score is a risk scoring method for evaluating lesion complexity based on anatomical characteristics of coronary angiographic lesions, but it does not incorporate relevant clinical factors. And the SYNTAX II score, a new score based on the SYNTAX score, which includes the presence or absence of an unprotected left main lesion. It is validated as a 4‐year mortality rate. However, the above scores and recommendations are derived from European and American populations. And most of the participants are men. In light of this, it is imperative to identify reliable prognostic predictions and individualized probability stratifications of AMI indicators in women.

An established predictor of cardiovascular disease is IR, which is a central characteristic of metabolic syndrome. Research has demonstrated that IR is associated with hypertension, dyslipidemia, diabetes mellitus, and a variety of other conditions that lead to cardiovascular disease [4, 5, 6]. Currently, however, when it comes to quantifying IR, the hyperinsulinemic euglycemic clamp (HIEC) is the gold standard. This method is complex, invasive, and time consuming [7]. A reliable surrogate marker for IR has recently been proposed: the TyG index. It incorporates data on fasting triglycerides (TG) and fasting plasma glucose (FPG). Previous studies have found that the TyG index is consistent with the hyperinsulinemic‐hyperglycemic clamp test and the homeostatic model assessment‐insulin resistance (HOMA‐IR) in the assessment of IR [8, 9, 10].

It has been proven that the TyG index is not a reliable predictor of outcome among patients with AMI, according to Luo et al., who conducted a cohort study on a total of 1092 patients. Within a year following PCI, a substantial and independent predictive predictor for severe adverse cardiovascular and cerebrovascular events was found in a study of patients with ST‐segment elevation myocardial infarction (STEMI) [11]. Subsequently, among the patients with non‐ST elevation acute coronary syndromes (NSTE‐ACS), Mao et al. discovered that the TyG index performed well for the prediction of major adverse cardiovascular outcomes [12]. It is disappointing that there is so little information on the correlation between TyG index and AMI risk in female patients.

It is unknown whether TyG index is associated with clinical outcomes and functions independently as a prognostic factor in women with AMI. Therefore, our study tested whether TyG index affects patient outcomes and functions independently as a prognostic factor.

## 2 Methods

### 2.1 Study Population

From January 2017 to December 2019, we conducted a retrospective cohort study at Fujian Provincial Hospital in China, enrolling consecutive women with type 1 MI based on the fourth universal definition of MI [13]who undergoing coronary angiography. The exclusion criteria were the following: (I) lack of data on fasting glucose, triglycerides, coronary angiography; (II) cardiogenic shock, left ventricular ejection fraction (LVEF) < 30%; (III) severe hepatic and renal failure; (IV) cancer with a life expectancy of less than 1 year; (V)suspected familial hypertriglyceridemia (plasma triglycerides≥ 500 mg/dL). Our analysis ultimately included 320 patients who met the inclusion criteria.

As this was a retrospective study, no written informed consent was necessary. The research was sanctioned by the hospital's ethics commission in Fujian Province. Everything was carried out in accordance with the Declaration of Helsinki of 1964 and its revisions.

### 2.2 Data Collection and Definitions

Based on hospital records, we collected baseline data on the study population's characteristics, like demographics, medical history, first recorded vitals on admission, and echocardiographic report. After an overnight fast of at least 8 h, venous blood samples were taken for the laboratory analysis (e.g., serum levels of FPG an TG) which were at the clinical laboratory using the standard methods. Additionally, two independent angiographers reviewed the coronary angiography evaluation. Following is how the TyG index was computed: ln [fasting TG (mg/dL) × FPG (mg/dL)/2].

### 2.3 Follow‐Up and Study Endpoint

At each 6‐month period following AMI, patients, and their families were followed up in outpatient clinics or via telephone interview. This medical documentation and telephone interviews served as the source of follow‐up data (Table 4).

The primary endpoint of this trial was major adverse cardiovascular and cerebrovascular events (MACCEs), which included all‐cause mortality, myocardial infarction, repeat revascularization, rehospitalization for heart failure, and stroke.

### 2.4 Statistical Analysis

Statistics were analyzed using SPSS 26.0 (SPSS, Chicago, IL), and data visualizations were performed with the R programming language (R Core Team, 2022). Continuous variables were expressed as mean ± SD or median (inter‐quartile range) and checked by Kolmogorov–Smirnov test for normality; Categorical variables were shown in the form of frequency distributions and rate proportions. It was determined which variables to compare between groups in accordance with their types. The correlation between TyG and other indicators was evaluated by using Spearman's correlation test for continuous variables and the Mann–Whitney *U* test for categorical variables. The optimal cutoff value for the TyG for an adverse clinical outcome was determined through the analysis of receiver operator characteristic (ROC) curves. This test was evaluated for its accuracy using the area under the curve (AUC) and 95% confidence interval (CI). Kaplan–Meier survival curves were analyzed, and log‐rank tests were performed to see whether there were significant variations in survival times between the groups. Possible predictors were identified by univariate analysis, and parameters with *p* < 0.10 from univariate analysis or cardiovascular risk factors identified by the study were included in a multivariate Cox proportional hazards model for identing independent prognostic factors, and the hazard ratio (HR) and 95% CI were further derived. Missing data on covariate were handled using a missing indicator approach; missing data on outcomes were handled using pairwise deletion. For this study, a significance level of two‐sided *p* < 0.05 was used.

## 3 Results

### 3.1 Baseline Characteristics of Patients

**Chart 1:**
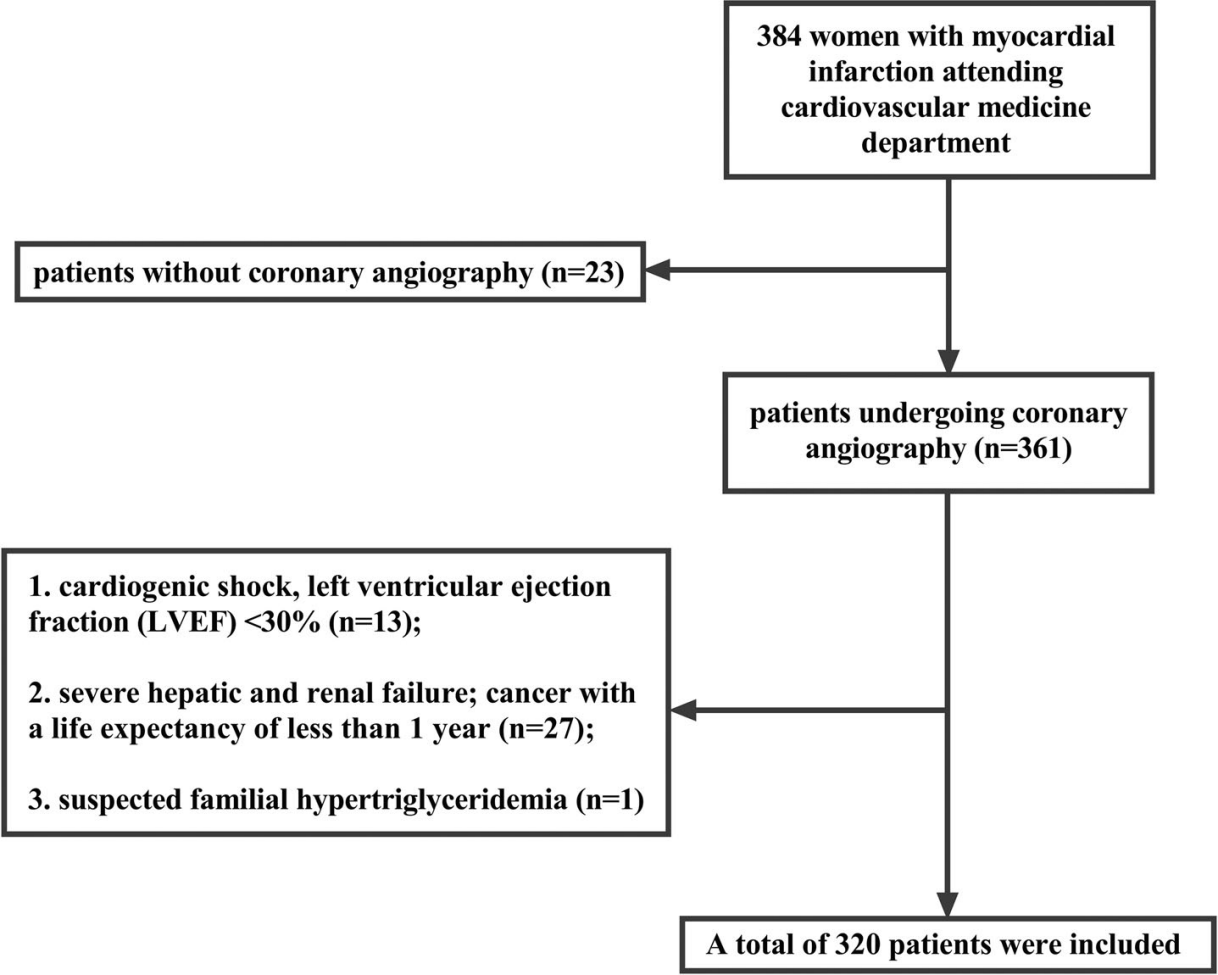
A flow chart of specific criteria for inclusion and exclusion.

Table 1 summarizes the clinical characteristics of all 320 patients participating in this study. All 320 women with the median age of 69 years (range: 30–91 years) were enrolled (Chart 1) and were divided into the low TyG group (*n* = 224) and the high group (*n* = 96). According to epidemiologic surveys, women are usually older at the time of their first AMI, with a mean age of 71.8 years than men, similar to the present study [3]. There was a higher likelihood of diabetes, LAD lesion, high levels of HDL cholesterol, hemoglobin A1c (HbA1C), and urea N in patients with high TyG indexes. Tissue thrombus aspiration and stent implantation were both more common in women with a higher TyG index (all *p* < 0.05).

**Table 1:** Baseline clinical characteristics of patients stratified by the optimal cutoff point of TyG index.

Variable	Total population (*n* = 320)	Lower TyG index (<9.3; *n* = 224)	Higher TyG index (≥9.3; *n* = 96)	*p*
Age, years	69.00 (62.00–74.50)	69.00 (62.50–75.00)	68.00 (62.00–74.00)	0.323
SBP, mmHg	131.00 (114.50–147.00)	130.00 (114.00–147.00)	133.00 (116.00–148.00)	0.499
DBP, mmHg	74.00 (66.00–83.00)	73.00 (65.50–80.50)	76.00 (67.00–86.50)	0.088
heart rate, bpm	75.00 (68.00–82.00)	74.00 (68.00–80.00)	78.00 (69.00–88.50)	0.001
Killip Class, *n* (%)				0.675
1	187 (58.44)	132 (70.59)	55 (29.41)	
2	97 (30.31)	69 (71.13)	28 (28.87)	
3	20 (6.25)	14 (70.00)	6 (30.00)	
4	16 (5.00)	9 (56.25)	7 (43.75)	
Menopause, *n* (%)	298 (93.13)	209 70.13)	89 (29.87)	0.847
Emergency admission	193 (60.31)	138 (71.50)	55 (28.50)	0.470
Initial diagnosis				0.363
STEMI	118 (36.88)	79 (66.95)	39 (33.05)	
NSTEMI	202 (63.13)	145 (71.78)	57 (28.22)	
Medical history, *n* (%)				
Hypertension	224 (70.00)	152 (67.86)	72 (32.14)	0.201
Diabetes	93 (29.06)	49 (52.69)	44 (47.31)	< 0.001
Coronary artery disease	26 (8.13)	16 (61.54)	10 (38.46)	0.326
Stroke	10 (3.13)	9 (90.00)	1 (10.00)	0.292
Atrial fibrillation	9 (2.81)	7 (77.78)	2 (22.22)	0.729
Post Hysterectomy	28 (8.75)	21 (75.00)	7 (25.00)	0.546
Laboratory results				
Albumin, g/L	39.00 (36.00–42.00)	39.00 (36.00–42.00)	39.00 (37.00–42.00)	0.370
LDL‐C, mmol/L	2.99 (2.33–3.89)	2.94 (2.34–3.68)	3.09 (2.31–4.26)	0.324
HDL‐C, mmol/L	1.08 (0.89–1.31)	1.14 (0.95–1.36)	0.95 (0.78–1.20)	<0.001
TG, mg/dL	133.30 (98.76–178.48)	116.03 (90.79–145.70)	201.06 (168.73–286.98)	<0.001
FPG, mg/dL	110.70 (93.96–142.65)	100.26 (90.27–120.24)	162.54 (117.81–213.39)	<0.001
Creatinine, μmol/L	65.00 (55.00–79.00)	65.00 (55.00–78.00)	65.00 (56.00–80.00)	0.686
Urea N, mmol/L	5.00 (4.10–6.40)	4.80 (4.00–6.15)	5.30 (4.20–7.00)	0.019
Na+, mmol/L	141.00 (138.00–142.00)	141.00 (139.00–143.00)	139.00 (138.00–141.00)	<0.001
K+, mmol/L	4.10 (3.90–4.40)	4.10 (3.90–4.40)	4.10 (3.80–4.40)	0.640
HbA1c, %	6.25 (5.80–7.65)	6.05 (5.70‐6.65)	7.75 (6.30–9.70)	< 0.001
TNI, ng/mL	5.81 (1.16–26.58)	6.32 (1.18–26.16)	4.05 (1.09–34.44)	0.971
NT‐ProBNP, pg/mL	1356.0 (583.50–2980.0)	1329.0 (607.90–3173.0)	1414.5 (473.30–2813.0)	0.802
LVEF, %	57.00 (52.00–60.00)	57.00 (52.50–60.00)	57.00 (52.00–60.00)	0.592
*Angiographic data*				
Diseased vessel number			0.063	
0	32 (10.00)	29 (90.63)	3 (9.38)	
1	74 (23.13)	51 (68.92)	23 (31.08)	
2	60 (18.75)	41 (68.33)	19 (31.67)	
3	154 (48.13)	103 (66.88)	51 (33.12)	
LM disease, *n* (%)	26 (8.13)	21 (80.77)	5 (19.23)	0.211
LAD disease, *n* (%)	250 (78.13)	167 (66.80)	83 (33.20)	0.018
LCX disease, *n* (%)	196 (61.25)	130 (66.33)	66 (33.67)	0.071
RCA disease, *n* (%)	205 (64.06)	137 (66.83)	68 (33.17)	0.098
SYNTAX score	2.25 (0.00–15.00)	2.75 (0.00‐15.00)	1.00 (0.00–13.00)	0.695
Procedural results				
Stent implantation, *n* (%)	15 (4.69)	6 (40.00)	9 (60.00)	0.038
Number of stents	1.00 (0.00–2.00)	1.00 (0.00–2.00)	1.00 (1.00–2.00)	0.361
Stent diameter, mm	2.75 (2.50–3.00)	2.75 (2.50–3.00)	2.75 (2.50–3.00)	0.318
Stent length, mm	33.00 (23.00–36.00)	33.00 (23.00–36.00)	29.00 (23.00–36.00)	0.633
Thrombus aspiration, *n* (%)	15 (4.69)	6 (40.00)	9 (60.00)	0.017
GPIIb/IIIa antagonists, *n* (%)	15 (4.69)	6 (40.00)	9 (60.00)	0.861
Second operation, *n* (%)	20 (6.25)	12 (60.00)	8 (40.00)	0.314

*Note:* Data are presented as median (interquartile range), or *n* (%).

Abbreviations: DBP, diastolic blood pressure; FPG, fasting plasma glucose; HbA1c, Hemoglobin A1c; HDL‐C, high‐density lipoprotein cholesterol; LAD, left anterior descending artery; LCX left circumflex artery; LDL‐C, low‐density lipoprotein cholesterol; LM, left main artery; LVEF, left ventricular ejection fraction; NSTEMI, non‐ST‐segment elevation myocardial infarction; RCA right coronary artery; SBP, systolic blood pressure; STEMI, ST‐segment elevation myocardial infarction; TG, triglycerides; TyG index, triglyceride‐glucose index; TNI, troponin I.

Youden index determined that 9.3 was the best possible cutoff for the TyG index. Then patients were divided into the low TyG group (TyG index < 9.3) and the high TyG group (TyG index ≥ 9.3) for further analyses. The baseline clinical features of the 320 patients in the study are shown in Table 1.

### 3.2 Correlation Between TyG Index and Cardiovascular Risk Factors

**Figure 1:**
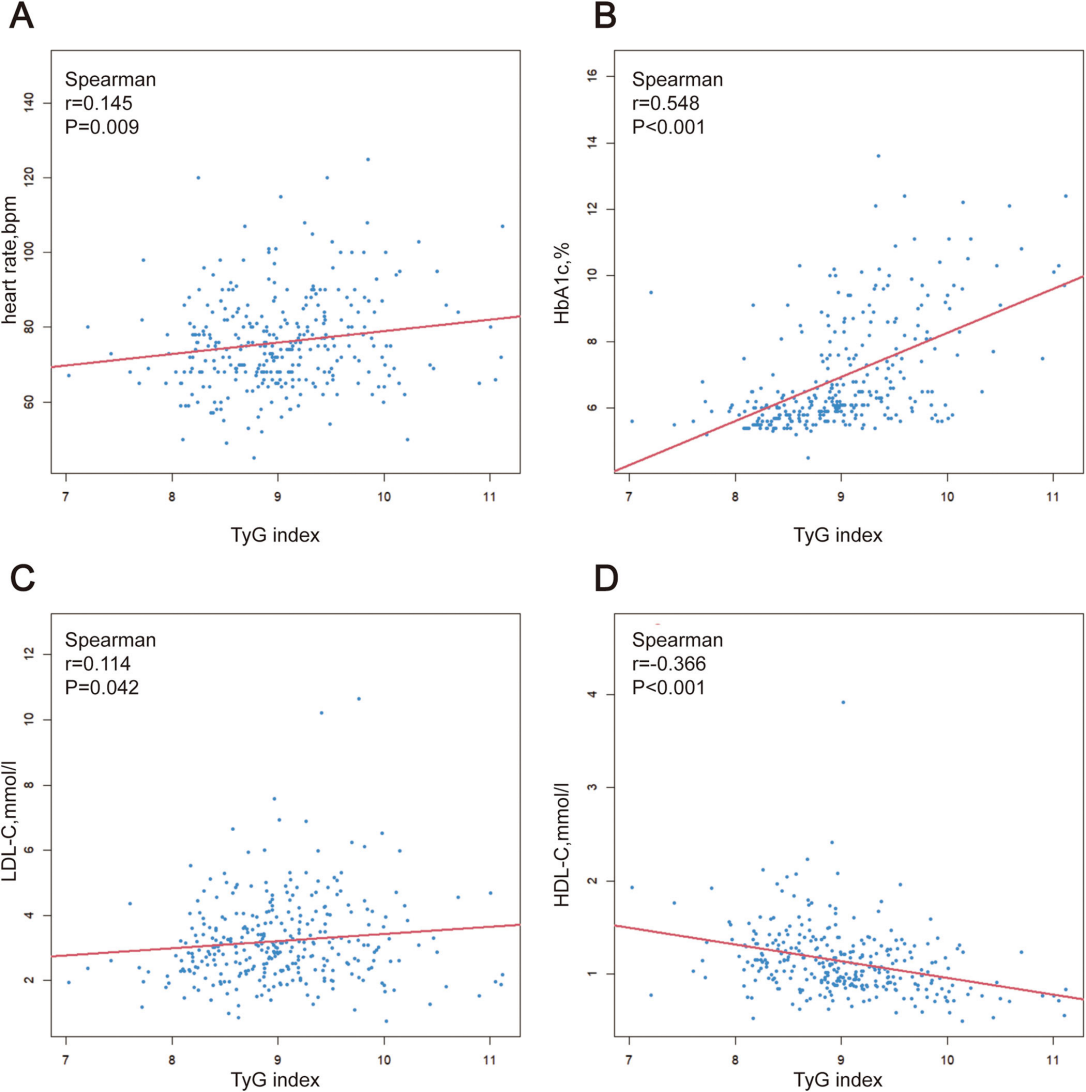
Correlations between TyG index and (A) heart rate, (B) HbA1c, (C) LDL, (D) HDL were evaluated via Spearman's correlation analysis in the whole patients. The *p* value < 0.05 is considered as significant. Spearman's correlation coefficients (abbreviated *r*) range from −1 to +1. The sign of the coefficient indicates whether it is a positive or negative monotonic relationship. A positive correlation means that as one variable increases, the other variable also tends to increase. A negative correlation signifies that as one variable increases, the other tends to decrease. Values close to −1 or +1 represent stronger relationships than values closer to zero. HbA1c, Hemoglobin A1c; HDL‐C, high‐density lipoprotein cholesterol; LDL, low‐density lipoprotein cholesterol; TyG index, triglyceride‐glucose index.

Table 2 shows that TyG index had a weak but significantly positive relationship with hypertension (*r* = 0.118, *p* < 0.05) and diabetes (*r* = 0.293, *p* < 0.001), respectively. Additionally, as presented in Figure 1, the TyG index showed a positive correlation with heart rate (*r* = 0.145, *p* = 0.009), low‐density lipoprotein cholesterol (LDL‐C, r = 0.114, *p* = 0.042), HbA1c (r = 0.548, *p* < 0.001) and number of diseased vessels. Our research also revealed that the TyG index was negatively correlated with high‐density lipoprotein (HDL‐C) (*r* = −0.366, *p* < 0.001). Unfortunately, the TyG index and the SYNTAX scores do not correlate with one another in any meaningful way (Table 2).

**Table 2:** Correlation between TyG index and cardiovascular risk factors.

Variable	*r*	*p*
Hypertension	0.118	0.035
Diabetes	0.293	<0.001
heart rate, bpm	0.145	0.009
LDL‐C, mmol/L	0.114	0.042
HDL‐C, mmol/L	−0.366	<0.001
HbA1c, %	0.548	<0.001
TNI, ng/mL	0.037	0.513
NT‐ProBNP, pg/mL	−0.018	0.751
LVEF, %	−0.015	0.807
SYNTAX score	0.018	0.745
Number of diseased vessels	0.133	0.018
Number of stents	0.101	0.072
Thrombus aspiration	0.082	0.145
Stent implantation	0.147	0.008

*Note:* Spearman's correlation coefficients (abbreviated *r*) range from −1 to +1. The sign of the coefficient indicates whether it is a positive or negative monotonic relationship. A positive correlation means that as one variable increases, the other variable also tends to increase. A negative correlation signifies that as one variable increases, the other tends to decrease. Values close to −1 or +1 represent stronger relationships than values closer to zero.

Abbreviations: DL‐C, low‐density lipoprotein cholesterol; HbA1c, Hemoglobin A1c; HDL‐C, high‐density lipoprotein cholesterol; LVEF, left ventricular ejection fraction; TNI, troponin I; TyG index, triglyceride‐glucose index; L.

### 3.3 The Prognostic Significance of TyG Index

**Figure 2:**
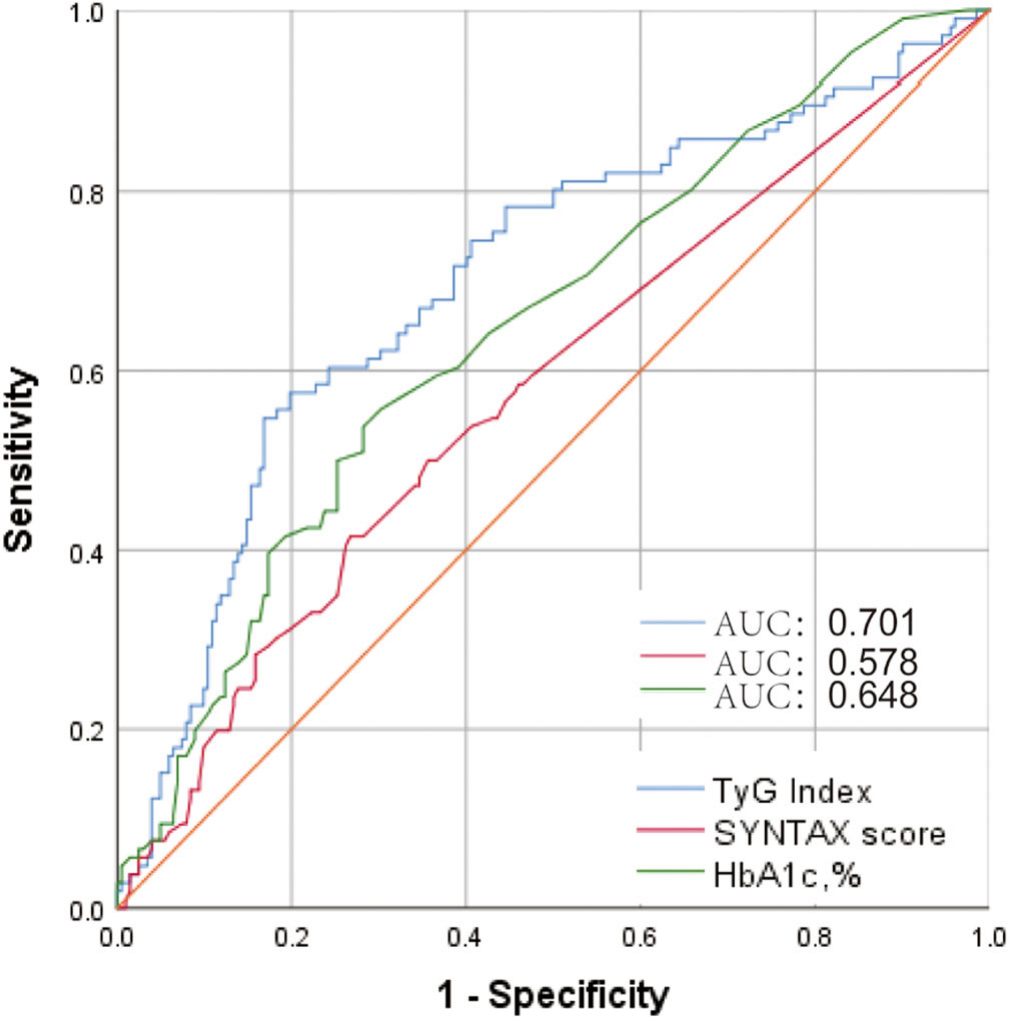
The receiver operating characteristic (ROC) curves of the TyG index, SYNTAX score and HbA1c. The area under ROC curves (AUCs) of the TyG index, SYNTAX score and HbA1c are 0.701,0.578 and 0.648. HbA1c, hemoglobin A1c; TyG index, triglyceride‐glucose index.

The median duration of follow‐up was 34.37 months. There were 111 patients who were hospitalized for MACCEs, 15 of whom died of all causes. Among the remaining 96 patients, 34 were readmitted after discharge for heart failure, 27 suffered myocardial infarction, 22 underwent revascularization, and 13 were hospitalized for stroke. Based on ROC curve analysis, the AUC was 0.701 (Figure 2).

**Figure 3:**
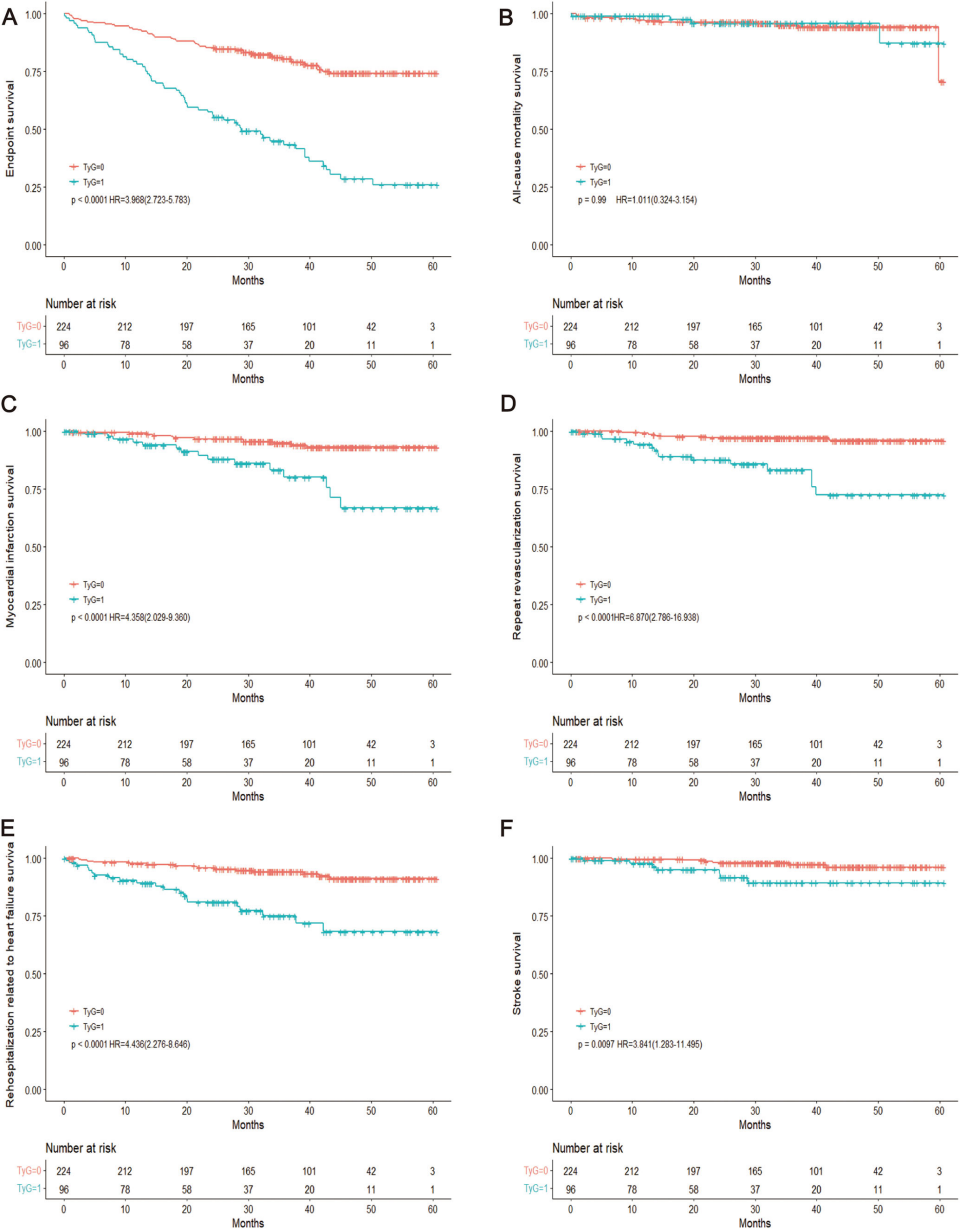
Survival outcomes in women with acute myocardial infarction stratified by TyG index. (A) Endpoints survival, (B) all‐cause mortality survival, (C) myocardial infarction survival, (D) repeat revascularization survival, (E) rehospitalization related to heart failure survival, and (F) stroke survival between low and high TyG index. The *p* value < 0.05 is considered as significant. TyG index, triglyceride‐glucose index; HR, hazard ratio. TyG=1: A high TyG index, TyG=0: A low TyG index.

**Figure 4:**
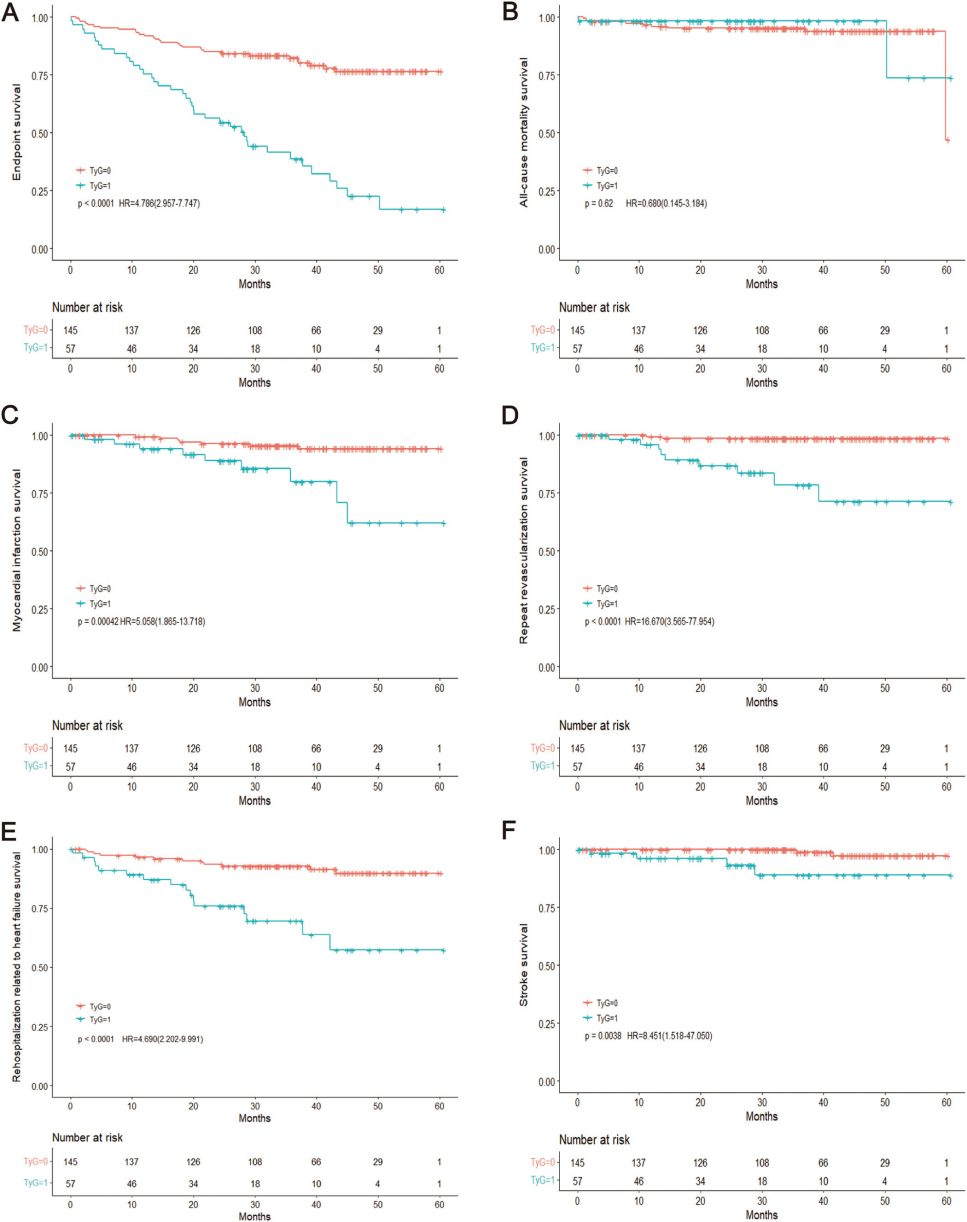
Survival outcomes in women with ST‐segment elevation myocardial infarction stratified by TyG index. (A) Endpoints survival, (B) all‐cause mortality survival, (C) myocardial infarction survival, (D) repeat revascularization survival, (E) rehospitalization related to heart failure survival, and (F) stroke survival between low and high TyG index. The *p* value < 0.05 is considered as significant. HR, hazard ratio; TyG index, triglyceride‐glucose index. TyG=1: A high TyG index, TyG=0: A low TyG index.

**Figure 5:**
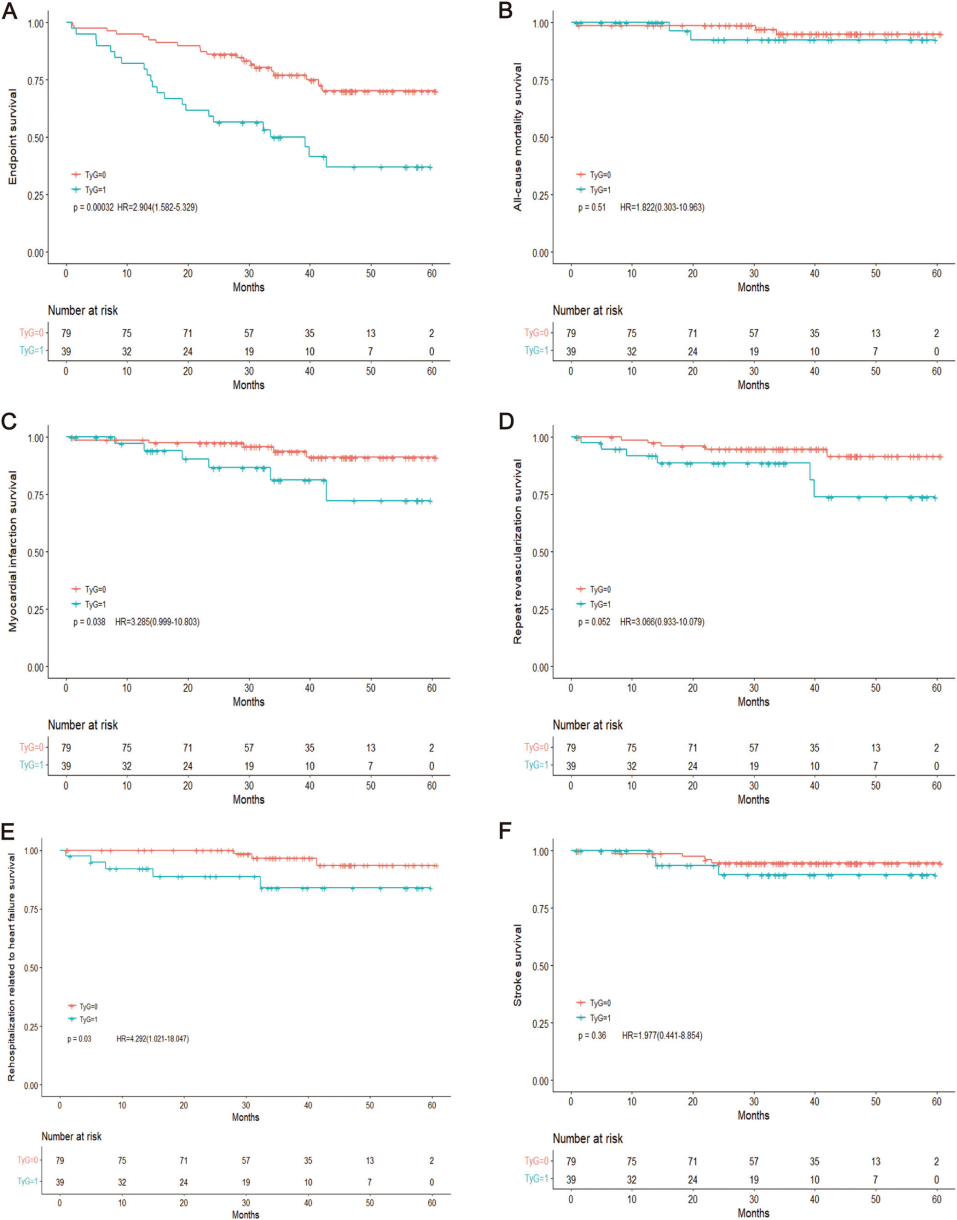
Survival outcomes in women with non‐ST‐segment elevation myocardial infarction stratified by TyG index. (A) Endpoints survival, (B) all‐cause mortality survival, (C) myocardial infarction survival, (D) repeat revascularization survival, (E) rehospitalization related to heart failure survival, and (F) stroke survival between low and high TyG index. The *p* value < 0.05 is considered as significant. HR, hazard ratio; TyG index, triglyceride‐glucose index. TyG=1: A high TyG index, TyG=0: A low TyG index.

A higher TyG index was associated with a considerably increased risk of myocardial infarction, as seen by the Kaplan‐Meier curve when comparing individuals with lower and higher TyG indices (*p* < 0.001), repeat revascularization(*p* < 0.001), rehospitalization related to heart failure(*p* < 0.001), stroke(*p* = 0.01) (Figure 3). In addition, we examined the prognostic value of TyG index by stratifying the data by the level of diagnosis in a subgroup analysis. Similarly, patients with a higher TyG index, in both women with STEMI and the general population, had a substantially greater prevalence of poor cardiovascular events (*p* < 0.001, Figure 4) and women with non‐STEMI (NSTEMI) (*p* < 0.001, Figure 5).

**Figure 6:**
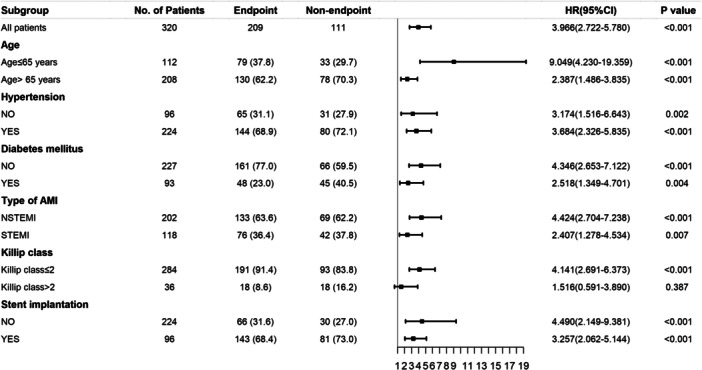
Cox proportional hazards analysis evaluating prognostic implication of TyG index in various stratifications. The *p* value < 0.05 is considered as significant. TyG index, triglyceride‐glucose index.

Prediction of adverse cardiovascular and cerebrovascular events was achieved using a risk stratification model. Table 3 illustrates univariate Cox regression analyses demonstrating the presence of significant prognostic factors such as age, heart rate on admission, HDL‐C, HbA1c, NT‐ProBNP, LVEF, SYNTAX score, diabetes, left circumflex artery disease, and TyG index as continuous or categorical variables. (*p* = 0.022, *p* = 0.025, *p* = 0.032, *p* < 0.001, *p* < 0.001, *p* = 0.002, *p* = 0.011, *p* < 0.001, *p* = 0.036, *p* < 0.001and *p* < 0.001 respectively). All these clinical characteristics were further investigated in a multivariate Cox regression analysis. After adjusting for potential confounding risk factors including TyG index as a categorical variable (HR: 4.292, 95% CI: 2.784–6.616, *p* < 0.001), NT‐ProBNP (HR: 1.001, 95% CI: 1.001–1.002, *p* = 0.001), left main artery disease (HR: 2.425, 95% CI: 1.235–4.761, *p* = 0.010) remained significant independent predictor of MACCEs. Moreover, patients were further stratified based on age, history of hypertension and diabetes, type of AMI, Killip class, and undergoing stent implantation. The greater the TyG index, the more strongly the MACCEs were correlated with it, except in patients with Killip class > 2 (Figure 6).

**Table 3:** Predictors of composite MACCEs.

Variable	Univariate analysis		Multivariate analysis	
	HR (95% CI)	*p*	HR (95% CI)	*p*
TyG index as a categorical variable	3.966 (2.722–5.780)	<0.001	4.292 (2.784–6.616)	<0.001
TyG index as a continuous variable	2.223 (1.712–2.887)	<0.001	2.223 (1.712–2.887)	
Age, years	1.024 (1.003–1.044)	0.022		
SBP, mmHg	1.003 (0.994–1.011)	0.557		
DBP, mmHg	1.003 (0.989–1.018)	0.650		
Heart rate, bpm	1.016 (1.002–1.030)	0.025		
Menopause	1.340 (0.589–3.052)	0.485		
Emergency admission	1.111 (0.758–1.627)	0.590		
Initial diagnosis	1.028 (0.700–1.509)	0.888		
Medical history				
Hypertension	1.121 (0.741–1.698)	0.588		
Diabetes	1.978 (1.354–2.891)	<0.001		
Coronary artery disease	1.189 (0.621–2.278)	0.601		
Stroke	0.819 (0.260–2.581)	0.734		
Atrial fibrillation	1.746 (0.712–4.284)	0.223		
Laboratory results				
Albumin, g/L	0.979 (0.937–1.023)	0.344		
LDL‐C, mmol/L	1.070 (0.920–1.245)	0.378		
HDL‐C, mmol/L	0.501 (0.266–0.942)	0.032		
TG, mg/dL	1.003 (1.002–1.005)	<0.001		
FPG, mg/dL	1.007 (1.004–1.009)	<0.001		
HbA1c, %	1.247 (1.133–1.372)	<0.001		
TNI, ng/mL	1.001 (0.999–1.002)	0.263		
NT‐ProBNP, pg/mL	1.000 (1.000–1.000)	<0.001	1.001 (1.0005–1.002)	0.001
LVEF, %	0.963 (0.940–0.987)	0.002		
Angiographic data				
LM disease	1.760 (0.986–3.141)	0.056	2.425 (1.235–4.761)	0.010
LAD disease	1.454 (0.887–2.384)	0.138		
LCX disease	1.537 (1.030–2.296)	0.036		
RCA disease	1.022 (0.694–1.506)	0.913		
SYNTAX score	1.019 (1.004–1.033)	0.011		
Procedural results				
Stent implantation	1.257 (0.826–1.913)	0.285		
Number of stents	1.019 (0.883–1.176)	0.800		
Stent diameter	0.965 (0.660–1.411)	0.855		
Stent length	1.000 (0.972–1.028)	0.996		
Thrombus aspiration	1.974 (0.998–3.904)	0.051		
GPIIb/IIIa antagonists	0.944 (0.596–1.494)	0.805		
Second operation	0.486 (0.179–1.319)	0.157		
Duration of the procedure	1.002 (0.996–1.009)	0.444		

Abbreviations: DBP, diastolic blood pressure; FPG, fasting plasma glucose; HbA1c, Hemoglobin A1c; HDL‐C, high‐density lipoprotein cholesterol; HR, hazard ratio; LCX, left circumflex artery; LAD, left anterior descending artery; LDL‐C, low‐density lipoprotein cholesterol; LVEF, left ventricular ejection fraction; LM, left main artery; MACCEs, major adverse cardiovascular and cerebrovascular events; RCA, right coronary artery; SBP, systolic blood pressure; TG, triglycerides; TNI, troponin I; TyG index, triglyceride‐glucose index.

**Table 4:** Follow‐up every 6 months after discharge.

Follow‐up time (month)	Cumulative number of persons with MACCEs	Cumulative MACCEs incidence
6	21	7%
12	36	12%
18	56	18%
24	74	24%
30	118	38%
36	173	55%
42	211	67%
48	254	80%
54	287	90%
60	316	99%
＞60	320	100%

Abbreviation: MACCEs, major adverse cardiovascular and cerebrovascular events.

## 4 Discussion

Until this study was conducted, the TyG index had never been evaluated on female patients suffering from AMI for prognostic purposes. There was a significant detrimental effect of a higher TyG index on the prognosis of female patients, regardless of whether they had STEMI or non‐STEMI. Even after adjusting for traditional cardiovascular risk factors, TyG index ≥ 9.3 was still a reliable predictive factor for MACCEs. The TyG score was demonstrated to be a significant predictor of mortality in female patients with AMI.

While comprehensive management has improved the prognosis of patients with myocardial infarction, some patients remain at high risk of poor outcomes even after receiving dual antiplatelet therapy, intensive lipid reduction, and coronary revascularization, as it has been known for some time [14]. Intriguingly, prognosis is greatly influenced by the underlying mechanism of AMI, which differs depending on gender. There is evidence that female patients are more likely to spend longer in the hospital and die more often than male patients [15, 16]. In fact, we found that approximately 1/3 of the women had an adverse outcome during the follow‐up in current study. Women with their first myocardial infarction may tend to delay admission to the hospital after onset of symptoms, which may be explained by their older age and their history of myocardial infarction [3]. Moreover, the scientific evidence supports AMI events among women may more likely to be due to plaque ulceration, plaque erosion, and vasospasm rather than plaque rupture [17, 18]. Therefore, it is important to better the health of this group by determining what variables may contribute to the worse prognosis experienced by female patients with AMI.

When insulin‐sensitive tissues including skeletal muscle, adipose tissue, the liver, and the pancreas are unable to respond normally to insulin, a condition known as IR occurs [19]. Inflammatory reactions are characterized by a complex network of glucose and lipid metabolism, involving an important role for the inflammatory cascade [20]. Some research suggests that people with cardiovascular illness who also have IR have a worse prognosis [4, 5, 6]. Framingham Heart Study results found that IR was associated with thickened left ventricular wall in women without being associated with thickened wall in men [20, 21]. As a proxy test of IR, the TyG index is more convenient, inexpensive, and easily available than the HIEC, which is the gold standard but req. 1ires a lot of time and effort to complete. FPG and fasting TG are used to calculate TyG index, according to the formula. Studies have revealed that FPG reflects IR mainly from the liver, whereas TG reflects IR from the adipose. It follows that the TyG index has a high degree of correlation with IR, as it can serve as a dual‐purpose reflection of IR [22]. There has been evidence that The TyG index can be used to screen the population for IR, regardless of whether or not the person has diabetes. In terms of assessing IR, it is noteworthy that hyperinsulinemic euglycemic glucose clamp has a strong correlation with the TyG index and is highly correlated with the HOMA‐IR [23, 24].

Based on a systematic review, we discovered that patients with AMI who had a higher TyG index were at greater risk for developing cardiovascular complications. Moreover, the results were consistent in patients with STEMI and NSTEMI, with or without diabetes, and in patients who underwent percutaneous coronary intervention [11, 12, 22]. In spite of the numerous studies that have investigated the tie of the TyG index to different populations, very few studies have examined its correlation with females. An analysis of a subgroup of a observational study by Shao et al. indicates that for women with ACS, the TyG index is a far better predictor of MACE risk than it is for males [25]. Zou also revealed that the TyG index was independently associated with MACEs in female patients undergoing PCI with drug‐eluting stents, therefore these findings are consistent [26]. We included traditional cardiovascular risk factors such as hypertension, diabetes, and hyperlipidemia analysis of multivariate Cox models indicated that the TyG index was significantly associated with long‐term MACCEs after AMI in women and that this association remained significant after multivariate Cox regression was conducted. Furthermore, TyG index impacted worse prognosis for females with all types of AMI, STEMI and NSTEMI included. It can be concluded from these results that the TyG index was a reliable indicator of cardiovascular outcomes in women (AUC = 0.701), and may be a valuable tool for early identification of people at risk for poor outcomes. As reported in an earlier study, the TyG index predicted MACCE rates after intervention therapy in STEMI women with 0.654 AUC [11]. Furthermore, we explored the relationship between the TyG index and different subgroups of MACCEs (including all‐cause mortality, myocardial infarction, repeat revascularization, rehospitalization related to heart failure, stroke), to improve our insights into the predictive value of the TyG index for each adverse event. A subgroup analysis revealed no predictive value for all‐cause mortality for the TyG index, despite having a predictive value for MACCEs. It's a great study because we've extended the prognostic significance of TyG to a different group of women with AMI, totally confirming and complementing previous research.

The specific mechanism linking the TyG index to poor prognosis remains unknown; nevertheless, there are a number of viable hypotheses. To begin, the TyG index has been shown to strongly correlate with conventional cardiovascular risk variables in a number of investigations. Our investigation demonstrated a strong association between the TyG index and other risk factors, including hypertension and diabetes. It was shown that patients with higher TyG indices had a greater likelihood of having multiple, more serious, and complicated clinical problems. The findings were in correlation with those of Zhao et al. [22] Secondly, Atherogenesis is believed to be influenced by IR. Inflammatory response can be caused by IR, which changes glucose imbalances and changes lipid metabolism. A further consequence of IR is the dysfunction of the endothelium [27, 28]. Therefore, it is clear that in the current investigation, an increase in the TyG index was associated with a rise in LDL and the predominance of sick arteries. The TyG index did not correlate with SYNTAX scores, which quantify coronary lesion severity, as previously shown [12]. This may be due to the different plaque characteristics in women. Third, TyG index is significantly related to coagulation imbalance based on the results of this study. IR in AMI patients plays an important role in promoting local platelet activation and thrombin production [29, 30], resulting in a large thrombus burden. Perhaps this explains part of our finding that patients with a higher TyG index have a higher proportion of thrombus aspirations. Lastly, in addition to being important in cardiovascular remodeling, cardiac metabolism, and microcirculatory dysfunction, IR has also been considered as a significant contributor to MACCEs. Myocardial oxygen consumption and energy production can be imbalanced because of IR, which increases sympathetic activity and activates the renin‐angiotensin‐aldosterone system [4, 31, 32].

It has significant implications for clinical practice that for AMI females, the TyG index is a predictor of unfavorable outcomes. The TyG index is a quick and straightforward technique to determine whether patients have a greater probability of experiencing negative consequences, which is calculated from two laboratory data, FPS and TG. Thus, we can take more positive measures to improve outcomes in certain high‐risk patients, such as exercise, dietary management, or even medications. As an additional consideration, we believe that treatments for reducing IR may be an effective strategy for preventing cardiovascular events in AMI women with a higher TyG index. Only arguments are presented in the current research. There has to be more prospective randomized controlled studies done to prove its efficacy.

This study has three main limitations. First, the current research's limited sample size and the fact that it was an observational cohort study limited its applicability even more. Moreover, the levels of FPS and TG may be affected by other factors, which will affect the precision with which prognoses can be predicted. Even though it is essential to analyze trends in TyG index over time, we did not do so in our research. Instead, we focused on the connection between static changes in TyG index and long‐term prognosis. For confirmation of the preliminary findings of our study, it is necessary to conduct additional large‐scale prospective studies. The present study provides novel insight into comprehensive management of female patients with AMI despite these limitations, by examining the relationship between TyG index and prognosis.

## 5 Conclusion

The TyG index has been demonstrated to be a novel independent risk predictor for women with AMI, potentially capable of predicting their prognosis. Therefore, the TyG index will be useful for stratification of the risk of female patients with AMI in routine clinical settings. It is a convenient, inexpensive, and reliable tool. The results of this study require further validation through prospective studies.

## Author Contributions

**Xiao‐xia Qiu:** data curation, formal analysis, investigation, methodology, project administration, writing–original draft. **Yong‐li Chen:** data curation, formal analysis, investigation, methodology, validation, writing–original draft. **Xin‐kang Wang:** data curation, methodology, project administration, writing–review and editing. **Re‐hua Wang:** writing–review and editing.

## Ethics Statement

The study was approved by the ethics committee of Fujian Provincial Hospital. As directed by the Helsinki Declaration of 1964 and its amendments, ethical considerations were taken into account at every stage of the process.

## Conflicts of Interest

The authors declare no conflicts of interest.

## Transparency Statement

The lead author Xin‐kang Wang, Re‐hua Wang affirms that this manuscript is an honest, accurate, and transparent account of the study being reported; that no important aspects of the study have been omitted; and that any discrepancies from the study as planned (and, if relevant, registered) have been explained.

## Data Availability

Due to privacy considerations, the data sets used in this work are unavailable to the general public.
